# Response to: “Comment on ‘Three Calcium Hydroxyapatite‐Based Dermal Fillers Marketed in Mexico: Comparison of Particle Size and Shape Using Electron Microscopy’”

**DOI:** 10.1111/jocd.70985

**Published:** 2026-06-09

**Authors:** G. A. Sanchez Rico, S. B. A. Canto

**Affiliations:** ^1^ Oneline Beauty Clinic Cancún Mexico; ^2^ Centro de Investigación Científica de Yucatán Mérida Mexico

Dear Editor,

We thank the authors for their interest in our article, “Three Calcium Hydroxyapatite‐Based Dermal Fillers Marketed in Mexico: Comparison of Particle Size and Shape Using Electron Microscopy” [1]. We appreciate the opportunity to address the methodological points raised in the Letter to the Editor concerning the preparation procedures and interpretation of the scanning electron microscopy (SEM) findings presented in our study.

The purpose of this response is to clarify the analytical rationale, scope, and limitations of the methodological approach employed. Different preparation strategies may be appropriate depending on the specific analytical objective under investigation. We therefore consider this exchange a constructive opportunity to discuss methodological aspects relevant to the morphological characterization of calcium hydroxyapatite (CaHA)‐based dermal fillers.

## 1 Study Objective and Sample Preparation

A central issue raised relates to the preparation of CaHA samples prior to SEM analysis. In this context, it is important to distinguish between two distinct analytical objectives:

- Characterization of the filler formulation as commercially manufactured and clinically administered, and
- Characterization of isolated CaHA particles following separation from the carrier matrix.

Both approaches represent valid investigative strategies; however, they require different preparation protocols.

Our study aimed to examine the morphological characteristics of the fillers as commercially formulated, thereby reflecting the material configuration encountered in clinical practice. Accordingly, the preparation protocol was designed to minimize manipulation and avoid procedures intended to isolate CaHA particles, such as centrifugation or washing steps.

While centrifugation and washing are established methods for particle isolation, these procedures may introduce mechanical forces or physicochemical alterations that influence the morphology observed under SEM. When the analytical objective is to evaluate formulation‐level morphology, minimizing sample manipulation may reduce preparation‐induced artifacts.

## 2 Sample Handling and Potential Artifacts

Sample handling represents another relevant methodological consideration in SEM‐based analyses of injectable biomaterials. Additional processing steps may introduce contaminants or preparation artifacts that complicate image interpretation. For comparative evaluation of commercial formulations, simplified and standardized preparation procedures may therefore reduce methodological variability.

In the present study, the preparation protocol was designed to preserve the structural organization of the formulation with minimal intervention. Briefly, aliquots of the product were deposited directly onto the SEM substrate and allowed to air‐dry under controlled laboratory conditions prior to sputter coating with a conductive layer. Imaging was subsequently performed across magnification ranges appropriate for evaluating microsphere morphology.

We recognize that air‐drying may introduce preparation‐related effects, including aggregation or partial structural collapse. However, identical preparation conditions were applied to all samples in order to ensure methodological consistency across the products analyzed.

Transparent reporting of preparation parameters—including substrate type, drying conditions, sputter‐coating material and thickness, accelerating voltage, and magnification range—is essential to improve reproducibility in future comparative studies.

## 3 Interpretation of Particle Morphology

Differences in reported particle morphology across studies may arise from several factors, including variations in preparation protocols, imaging parameters, product batches, and interpretation criteria applied during analysis.

With respect to previously reported observations of fractured particles in certain CaHA‐based fillers, we note that fracture patterns were not commonly observed under the preparation conditions used in our study. This observation should not be interpreted as definitive evidence that particle fracture cannot occur, but rather as a finding specific to the experimental conditions employed. Discrepancies between studies may therefore reflect differences in preparation forces, imaging conditions, or batch variability [2].

## 4 Sampling Considerations

We acknowledge that the sampling strategy used for morphological evaluation represents a limitation of the study. The analysis included a limited number of SEM fields and particles per product, and the results should therefore be interpreted as an exploratory morphological comparison rather than a comprehensive statistical characterization of particle distributions.

Future investigations would benefit from more robust sampling frameworks, including analysis of multiple SEM fields per sample, randomized field selection, increased particle counts per field, and evaluation of multiple product batches. Such approaches would allow a more reliable assessment of particle size variability.

In addition, reporting complete particle size distributions—such as median values, interquartile ranges, and density plots—would provide a more comprehensive representation of morphological variability than mean particle size alone [3].

## 5 Scope of the Study

The objective of our study was to provide an initial morphological comparison of three CaHA‐based dermal fillers currently available in the Mexican market using a consistent SEM preparation protocol. The study was not designed to establish product superiority nor to directly extrapolate morphological findings to clinical outcomes.

Rather, the results were intended to contribute to the physicochemical characterization of these biomaterials and to highlight the need for additional research—including mechanistic and in vivo investigations—to better understand the relationship between particle morphology, formulation properties, and biological responses following injection [4, 5].

## 6 Transparency and Disclosures

The SEM analysis was conducted at an independent scientific research center without affiliation to the aesthetic medicine industry. Although I participate as a trainer for several aesthetic medicine companies, the present study did not receive industry funding. These professional relationships were disclosed in accordance with the transparency policies of the journal.

## 7 Concluding Remarks

Advances in the scientific understanding of injectable biomaterials rely on transparent methodological reporting and constructive academic dialogue. Differences in preparation protocols across studies highlight the importance of clearly defining analytical objectives and providing detailed methodological descriptions.

We hope these clarifications contribute to the ongoing discussion and encourage future studies employing standardized preparation procedures and more extensive sampling strategies to further characterize CaHA‐based dermal fillers. Attention to sample handling and contamination control is also relevant when interpreting biomaterial morphology and associated clinical safety outcomes [6].

## Conflicts of Interest

The authors declare no conflicts of interest.

## Data Availability

The data that support the findings of this study are available from the corresponding author upon reasonable request.
